# Immune Dysregulation at the Maternal–Fetal Interface Exacerbates Adverse Pregnancy Outcomes in an Inflammatory Arthritis Murine Model

**DOI:** 10.3390/biomedicines13061440

**Published:** 2025-06-11

**Authors:** Chenxi Yang, Wenjuan Li, Xinxin Liu, Zijun Ma, Jun Chen, Quan Gong, Zachary Braunstein, Xiaoquan Rao, Yingying Wei, Jixin Zhong

**Affiliations:** 1 Division of Rheumatology and Immunology, Department of Internal Medicine, Tongji Hospital, Tongji Medical College, Huazhong University of Science and Technology, Wuhan 430030, China; yangchenxi0409@163.com (C.Y.); 15893098210@163.com (W.L.); 13647161982@163.com (X.L.); 18995674552@163.com (Z.M.); 2 Hubei Clinical Research Center of Hypertension, Hubei Key Laboratory of Wudang Local Chinese Medicine Research, Sinopharm Dongfeng General Hospital, Hubei University of Medicine, Shiyan 442000, China; chenjun0121@126.com; 3 Department of Immunology, School of Medicine, Yangtze University, Jingzhou 434023, China; gongquan1998@163.com; 4 Division of Hematology, Department of Internal Medicine, James Comprehensive Cancer Center, The Ohio State University Wexner Medical Center, Columbus, OH 43210, USA; zbbraunstein@gmail.com; 5 Division of Cardiology, Department of Internal Medicine, Tongji Hospital, Tongji Medical College, Huazhong University of Science and Technology, Wuhan 430030, China; xqrao@tjh.tjmu.edu.cn; 6 Key Laboratory of Vascular Aging (HUST), Ministry of Education, Wuhan 430030, China

**Keywords:** inflammatory arthritis, pregnancy outcomes, SKG mice, T cells, inflammatory cytokines

## Abstract

**Objective**:**** Inflammatory arthritis (IA) has been linked to a number of adverse pregnancy outcomes (APOs), but the mechanisms linking IA-related immune dysregulation to compromised reproductive success remain poorly understood. This project will examine how IA affects pregnancy outcomes and alters the associated immune microenvironment using SKG (ZAP70^W163C^) mice, a mouse model that suffers from arthritis resembling human IA. **Methods:** IA was induced in SKG mice on a C57BL/6J background via mannan exposure. Wild-type C57BL/6 mice served as controls. Pregnancy rates, conception time, embryo resorption rates, and immune parameters (cytokine levels and splenic/lymph node/placental immune cell subsets) were analyzed. Joint pathology was evaluated via histology (HE is staining) and anti-CCP antibody levels. Flow cytometry was used to analyze immune populations within the spleen along with the associated lymphatic nodes. **Results:** Synovial hyperplasia, elevated anti-CCP, and systemic inflammation were all observed in IA mice. Compared to controls, IA mice demonstrated a reduced mating success rate, prolonged conception time, decreased pregnancy rates, and increased embryo resorption. IA mice showed elevated Th1/Th17 cytokines-IFN-γ, TNF-α, and IL-17, and an expansion of pro-inflammatory immune cells, including NK cells, CD11b+ myeloid cells, neutrophils, M1 macrophages, and Tc1, in the spleen/lymph nodes. Placental immune dysregulation featured increased NKT, NK, and CD4+ cell infiltration. Conversely, anti-inflammatory subsets, such as M2 macrophages and dendritic cells, were reduced. **Conclusions:** IA increased APOs and skewed the immune microenvironment toward a pro-inflammatory state dominated by Th1/Th17/Tc1 responses and cytotoxic cell activation. These findings highlight immune dysregulation as a key driver of IA-associated pregnancy complications, providing mechanistic insights for therapeutic intervention.

## 1. Introduction

Inflammatory arthritis (IA) represents a category of chronic joint disorders characterized by persistent inflammation, encompassing conditions such as rheumatoid arthritis (RA), psoriatic arthritis (PsA), and ankylosing spondylitis (AS) [1,2,3]. IA commonly affects female patients of reproductive age [4,5]. RA and AS rank among the most prevalent forms of IA. Patients with IA encounter distinct obstacles in preconception planning and gestational care due to potential risks from active disease states and therapeutic interventions [5].

Pregnancy poses significant challenges for women with IA, which is typically diagnosed during their reproductive years. Despite advances in medical care that greatly improve disease outcomes in women with IA [6,7,8], these patients remain at a greater risk of adverse pregnancy outcomes (APOs). Studies have shown that women with systemic lupus erythematosus (SLE) encounter a higher incidence of complications in comparison to the general population, including pre-eclampsia (PE), eclampsia (E), premature birth (PB), and intrauterine growth retardation (IGR) [9]. Similarly, women with AS are at higher risk of cesarean section [10] and premature birth [11]. Studies have shown that offspring of women with SLE, RA, and AS are more likely to be small for gestational age [6,12,13]. Furthermore, women with RA and SLE have a reduced likelihood of delivering live-born infants [14,15]. Although the incidence of pregnancy complications is less frequent in individuals with RA than in those with SLE, IGR and PE are slightly more likely in RA-affected pregnant women [7].

Spondyloarthritis (SpA) refers to a spectrum of chronic immune-mediated disorders affecting the axial and peripheral joints, encompassing AS, psoriatic arthritis (PsA), reactive arthritis, enteropathic arthritis linked to inflammatory bowel disease (IBD), and undifferentiated forms [16]. Restricted spinal and pelvic mobility in females with SpA may contribute to fetal head malposition, thereby elevating the risks of labor dystocia, assisted vaginal delivery, and cesarean section (CS). These obstetric challenges are further linked to elevated maternal–neonatal complications and mortality rates [17]. Moreover, higher SpA disease activity has demonstrated dose-dependent associations with adverse gestational outcomes [13,18,19].

RA can impact women during their reproductive years [20]. In recent decades, significant progress has been made in the treatment of RA, including the development of biological agents and Janus kinase (JAK) inhibitors that improve disease outcomes. Women with RA are prone to infertility, with 42% experiencing difficulty in conceiving for over 12 months [14]. High disease activity, as well as the use of high-dose corticosteroids and nonsteroidal anti-inflammatory drugs (NSAIDs), is associated with infertility and APOs. Additionally, some drugs, such as methotrexate, are associated with teratogenicity, highlighting the importance of providing preconception care in routine practice. Given that disease activity can worsen pregnancy outcomes and that RA patients are prone to infertility, providing preconception care in routine practice is crucial. Although pregnancy outcomes in RA patients are generally better than in SLE patients, they are still worse than in the general population [21]. Therefore, careful monitoring of APOs, medication adjustments, and evaluation of disease activity during pregnancy are essential.

Notably, 48–65% of patients experience improvement in RA disease activity during pregnancy [22], yet approximately half of them encounter disease deterioration postpartum [23]. This pattern is thought to be related to immune adaptations during pregnancy that help accommodate the developing fetus. Concurrently, the number of pregnancies among women with IA has steadily increased [6,24], paralleled by an elevated risk of APOs [25]. Among autoimmune diseases, SLE carries the highest risk of poor prognosis, including IGR, low birth weight (LBW), and increased risks of cesarean section [26]. Although active disease during pregnancy is known to increase the likelihood of LBW and preterm birth due to placental insufficiency, the association between active RA and conditions such as PE and gestational hypertension remains controversial [27]. In those suffering from active RA, elevated levels of inflammation-associated cytokines may cause placental insufficiency, miscarriage, IGR, or preterm birth [26,28,29,30,31,32,33,34]. The association between RA activity with the outcomes of pregnancy was further quantified in recent studies, showing that infants born to RA mothers face higher risks of stillbirth, being small for gestational age, and exhibiting a LBW, congenital abnormalities, type 1 diabetes, and asthma. High RA activity has also been linked to preterm birth and cesarean delivery [35].

Therefore, pregnancy and childbirth can be significant challenges for women with RA, as changes in the medication regimen, disease flare-ups, and immune dysregulation may negatively affect both maternal and fetal outcomes [36,37,38]. Optimal management of these patients is critical. Over the past decade, the improved use of disease-modifying antirheumatic drugs (DMARDs) and the development of novel biologics have advanced RA treatment considerably. With these therapeutic advances, ensuring a healthy, complication-free pregnancy has become a primary goal in managing pregnant women with RA [38]. Nonetheless, further clarification is needed regarding the potential adverse maternal and fetal outcomes associated with RA.

Genetic linkage analysis localized the Skg locus to a chromosome 1 region containing the Zap70 gene. Characterization of SKG mice revealed a pathogenic missense mutation in Zap70, specifically modifying the first residue of the C-terminal SH2 domain. Functional rescue through exogenous human ZAP70 expression completely abrogated arthritic manifestations, definitively establishing this mutation as the disease etiology [39]. Preclinical animal models serve as indispensable tools for dissecting molecular pathways and screening candidate therapeutics. Their utility is particularly pronounced in translational investigations of immune-mediated arthropathies [40]. Emerging studies indicate that the SKG (ZAP70^W163C^) mutation confers susceptibility to both SpA and RA in mice following β-glucan or mannan exposure, mediated by synergistic dysregulation of innate and adaptive immune pathways [41,42,43,44].

This study uses a genetic IA mouse model to examine how IA affects the outcomes of pregnancy as well as the immune microenvironment. Through systematic and quantitative investigation of maternal and fetal outcomes from an immunological perspective, our research seeks to provide valuable guidance for the monitoring and management of pregnant women with IA.

## 2. Materials and Methods

### 2.1. Mouse Model of IA

This study employed two groups. IA-prone SKG (ZAP70^W163C^) mice on a C57BL/6J background (*n* = 24) were generated by introducing a Crispr-Cas9-mediated W163C mutation in ZAP70 in association with Shanghai Nanmo Biotechnology Co., Ltd. IA induction consisted of a single dose of 20 mg mannan (M7504, Sigma, St. Louis, MO, USA) [42,44,45]. A total of 12 age-, sex-, and weight-matched female wild-type C57BL/6J mice served as controls. Pairing was conducted 14 days after IA induction. The sample size was determined based on preliminary experiments and power analysis. All procedures were approved by the Animal Ethics Committee in Tongji Hospital and carried out in an SPF barrier environment.

### 2.2. Mouse Model of IA Pregnancy

Female mice were caged with C57BL/6 males (one male paired with two females) to establish a pregnancy model 14 days after IA induction. The pairing was conducted between 5:00 and 6:00 p.m. Vaginal plugs were checked twice daily after pairing (at 8:30 a.m. and 3:00 p.m.). The detection of a vaginal plug was recorded as gestational day 0 (P0). When vaginal plugs were observed in female mice, males and females were separated into different cages. The mice were euthanized on gestational day 15 (P15).

### 2.3. Mouse Dissection

On day 15 of pregnancy, the pregnant mice were weighed and anesthetized with isoflurane (RWD, Jinan, Shandong), followed by euthanasia through CO_2_ inhalation. The uterus was exposed, and the number of viable and abnormal embryos was observed and recorded. The uterine wall was gently torn open at the uterine horn, and normal viable embryos adjacent to resorbed embryos were selected. The embryo and placenta unit was detached from the uterine wall, ensuring the integrity of the basal decidua. The embryo was gently separated from the placenta, and the fetal mouse and placenta were weighed. The spleen, mesenteric lymph nodes, axillary lymph nodes, and inguinal lymph nodes were removed, and the surrounding tissues were discarded.

### 2.4. Preparation of Single-Cell Suspensions from Spleen and Lymph Nodes

Single-cell suspensions were prepared by grinding the spleen or lymph nodes in a culture dish filled with sterile PBS (biosharp, Beijing, China). The suspension was then centrifuged at 300× *g* for 5 min. After centrifugation, the supernatant was discarded, and 1–2 mL of red blood cell lysis solution (Solarbio, Beijing, China) was added, mixed thoroughly, and incubated for 5 min. After adding an equal volume of PBS to stop the lysis of red blood cells, the suspension was then filtered through a 300-mesh filter and then subjected to centrifugation at 300× *g* for 5 min. Following that, the pellets were resuspended in PBS and analyzed by flow cytometry.

### 2.5. Digestion of Placentas and Preparation of Single-Cell Suspensions

Half of the excised placentas were placed in PBS. The placental tissue was ground on ice with gentle movements and then centrifuged at 400× *g* and 4 °C for 5 min. The supernatant was discarded, and 1 mL of Hank’s (biosharp, Beijing, China) digesting solution was added into each tube, which comprised 0.5 mg/mL Collagenase D (Merck, Mannheim, Germany), 1 mg/mL dispase (Merck, Mannheim, Germany), and 0.5 mg/mL DNase I (Solarbio, Beijing, China). The EP tube was sealed with a sealing film and inverted to fully resuspend the placental tissue in the digestion solution. It was then placed in a pre-warmed 37 °C shaking water bath for 30 min. After digestion, 1 mL of full culture medium was added to the EP tube to terminate the reaction. The tube was inverted, and the remaining steps were performed as quickly as possible. A Pasteur pipette was used to aspirate the mixed liquid, and the placenta single-cell suspension was filtered into an EP tube using a 70 µm filter. The collected filtrate was centrifuged at 400× *g*, 4 °C for 5 min. The supernatant was discarded, and 500 µL of red blood cell lysis buffer was added to the pellet. The mixture was thoroughly mixed and incubated at room temperature for 5 min. After lysis, 1 mL of 1× PBS was added to terminate the reaction. The sample was centrifuged at 400× *g* for a duration of 5 min at a temperature of 4 °C. The supernatant was discarded, and the pellet was resuspended in PBS for subsequent flow cytometry analysis.

### 2.6. Calculation of Embryo Resorption Rate


$$\mathrm{Embryo resorption rate}=\frac{\left(\mathrm{Number of resorbed embryos}\right)}{\left(\mathrm{Number of viable embryos + Number of resorbed embryos}\right)}\times 100\%$$

### 2.7. ELISA

Using an antibody-specific ELISA kit (CSB-EQ027743MO, CUSABIO, Wuhan, China), the concentrations of CCP in plasma were measured according to the instructions provided by the manufacturer.

### 2.8. CBA

To determine the expression levels of Th1/Th2/Th17 cytokines, the CBA detection kit (560485, BD, San Jose, CA, USA) was utilized in accordance with the directions provided by the manufacturer.

### 2.9. Hematoxylin-Eosin Staining

The joints were fixed in 4% paraformaldehyde for 72 h and then decalcified with EDTA solution (BIOSSCI, Wuhan, China) until fully decalcified. After dehydration and embedding, each section was subjected to staining using hematoxylin and eosin. Tissue sections were prepared using a microtome (Leica RM2255, Leica Biosystems, Nussloch, Germany). Serial sections of 4 μm thickness were obtained.

### 2.10. Flow Cytometry

Cells were subjected to staining with surface markers, including FVS-AF700 (564997, BD), CD45-BV785 (103149, Biolengend, San Diego, CA, USA), CD3-BV605 (100237, Biolengend), CD4-BB700 (566407, BD), CD8-ECD (562283, BD), NK1.1-BV605 (108735, Biolengend), CD11b-PE/CY7 (101216, Biolengend), CD25-PE (101904, Biolengend), CD44-BV421 (103040, Biolengend), CD62L-BV510 (104441, Biolengend), CD69-APC (104514, Biolengend), CD80-FITC (104706, Biolengend), MHCII-BV650 (107641, Biolengend), CD206-PE (141706, Biolengend), F4/80-BV421 (123132, Biolengend), Ly6G-BV510 (127633, Biolengend), and Ly6C-APC (128016, Biolengend). Staining of myeloid cell subsets was preceded by Fc receptor (FcR) blocking to eliminate non-specific binding. CD3 dNK cells were identified using CD3 and NK1.1. T cells were analyzed using CD3, CD4, and CD8. Macrophages were identified by the CD11b, F4/80, M1 (CD80), and M2 (CD206) markers. Th1/Th17 subtypes were further assessed by intracellular cytokine staining (IFN-γ for Th1, and IL-17 for Th17). CD4+ T cell activation was identified by the CD4, CD44, and CD62L markers. Dendritic cells, monocytes, and neutrophil cells were identified by the CD11b, CD11c, MHCII, Ly6G, and Ly6C markers. For intracellular labeling of IL-17A-APC and IFN-γ-BV480, cells were incubated with eBioscience™ Cell Stimulation Cocktail (with a protein transport inhibitor) (500×) (00-4795-73, Invitrogen, Carlsbad, CA, USA) for 4.5 h, fixed and permeabilized, and subsequently stained with IL-17A-APC and IFN-γ-BV480. The staining kits for transcription factors (Invitrogen, Carlsbad, CA, USA) were utilized in accordance with the protocol provided by the manufacturer. Analysis was performed using the CytoFLEX flow cytometer (Beckman, Miami, FL, USA), and the results were subsequently analyzed with FlowJo software (version 10.8.1).

### 2.11. Statistical Analysis

Normality was assessed using the Shapiro-Wilk test. A non-paired Student’s *t*-test was used for data that conformed to a normal distribution, and the non-parametric Mann–Whitney U test was used for data that did not conform to a normal distribution. All data were presented as medians (range). The value of *p* < 0.05 was considered statistically significant. Statistical analysis and graphing were performed using GraphPad Prism 10 (San Diego, CA, USA).

## 3. Results

### 3.1. Induction of IA in SKG Mice

**Figure 1:**
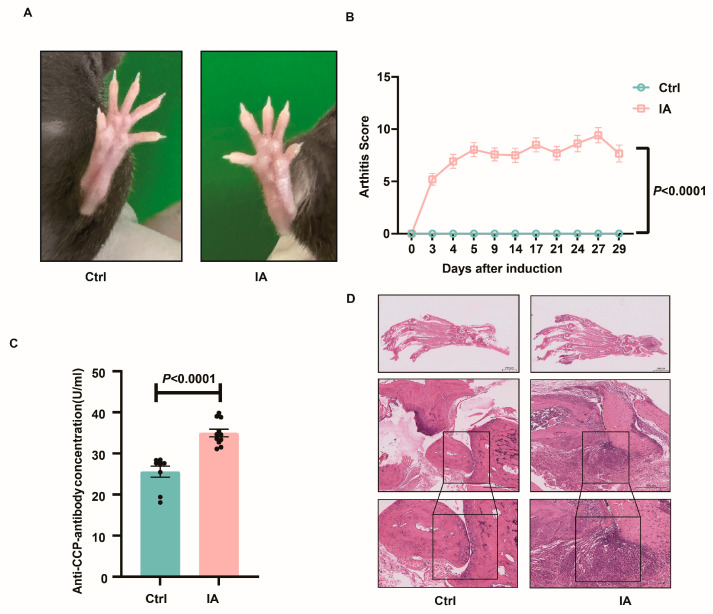
Establishment of the IA model. (**A**) Representative images of the paws after IA inductions. (**B**) Joint scores recorded before and after IA induction, showing significantly higher scores in the IA group compared to the Ctrl group. (**C**) ELISA quantification of plasma anti-CCP levels, demonstrating elevated expression in IA mice. (**D**) Histopathological examination of joint tissue by H&E staining confirms IA-associated joint pathology. Synovial hyperplasia and extensive inflammatory cell infiltration in the joint cavity of IA mice (black frame).

C57BL/6J-SKG female mice were intraperitoneally injected with 20 mg of mannan to induce the development of arthritis. Wild-type C57BL/6J mice were used as a control. We observed significant joint swelling in C57BL/6J-SKG female mice 3 days after induction, whereas no joint swelling was observed in the control group (Figure 1A). The degree of joint swelling was closely monitored, and joint scores were recorded according to the scoring criteria reported previously [46]. A notable rise in the arthritis score was observed in the IA group (*p* < 0.001) (Figure 1B). ELISA analysis of plasma revealed that anti-CCP antibody levels were substantially higher among the IA animals in comparison with the control (27.62 (22.50, 28.65) vs. 34.55 (32.94, 37.00), *p* < 0.001) (Figure 1C). In addition, histological examination revealed synovial hyperplasia and extensive inflammatory cell infiltration in the joint cavity of IA mice when contrasted with the control (Figure 1D). All these data indicate the successful establishment of the IA model.

### 3.2. APOs in the IA Group

**Figure 2:**
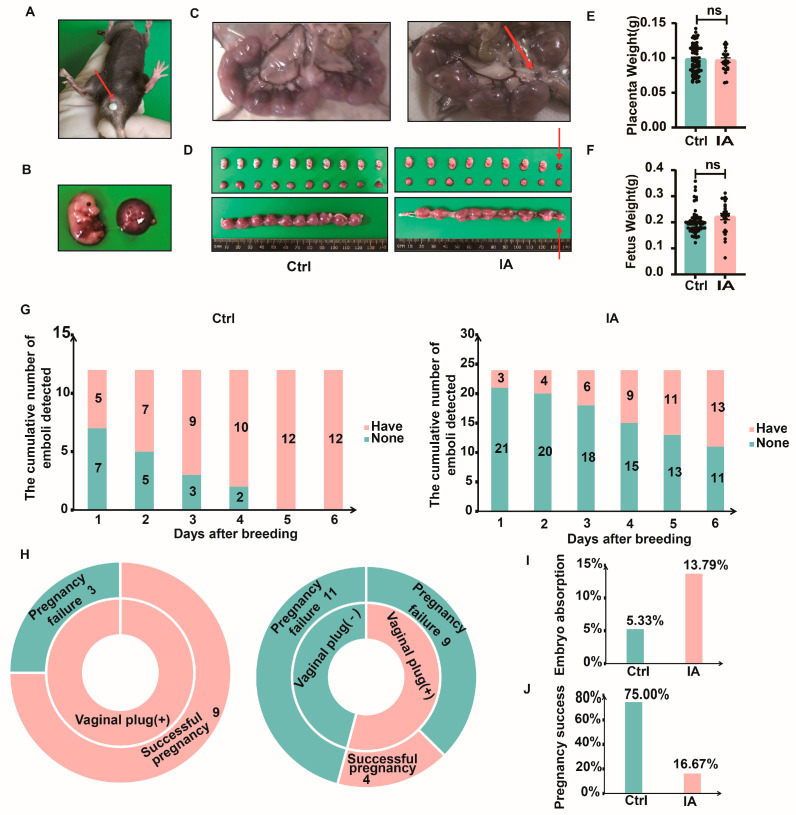
Establishment of the IA pregnancy model. (**A**) Representative image of a vaginal plug, indicating successful mating (red arrow). (**B**) Representative image showing the fetus, umbilical cord, and placenta. (**C**) Representative image showing the uterine morphology of Ctrl and IA mice on day 15 of pregnancy, with the red arrow indicating a dead/resorbed fetus surrounded by adipose tissue. (**D**) Representative image of the fetus and placenta from Ctrl and IA group pregnancies, with the red arrow indicating a dead/resorbed fetus surrounded by adipose tissue. (**E**) Graph comparing placenta weights between the Ctrl and IA groups. (**F**) Graph comparing fetal weights between the Ctrl and IA groups. (ns: not significant, *p* > 0.05) (**G**) Cumulative number of successful vaginal plugs observed in 12 female Ctrl and 24 female IA mice. (**H**) Summary of vaginal plug detection and pregnancy status in 12 female Ctrl mice and 24 female IA mice. (**I**) Graph comparing embryo resorption rates between the Ctrl and IA groups. (**J**) Graph comparing pregnancy success rates between the Ctrl and IA groups.

After 14 days of IA induction, the female mice were co-housed with C57BL/6 male mice at a 2:1 ratio (female/male). When a vaginal plug appeared in a female mouse, as shown by the red arrow (Figure 2A), mating was considered successful. In order to investigate the impact of IA on pregnancy outcomes, the mice were slaughtered on the 15th day of pregnancy (P15). Frequent embryo resorptions were observed in the IA group (Figure 2B–D). However, IA did not affect the weights of fetuses (0.19 (0.19, 0.21) vs. 0.23 (0.20, 0.25), *p* = 0.06) and placentas (0.10 (0.09, 0.10) vs. 0.10 (0.09, 0.10), *p* = 0.86) (Figure 2E,F). Continuous observation of the mating behavior showed that in the Ctrl group, all 12 mice successfully mated by the fifth day of cohabitation, while in the IA group, 11 out of 24 mice had successful plugs (Figure 2G). The success rate of vaginal plugs in the Ctrl group by day 6 was 12/12 (100%), which was substantially higher compared to the IA group at 13/24 (54.17%). To assess the pregnancy rates between the two groups, all 12 mice in the Ctrl group had successful vaginal plugs, with 9 mice becoming pregnant. In the IA group, 13 out of 24 mice had successful plugs, with 4 mice becoming pregnant (Figure 2H). The embryo resorption rate was 5.33% (4/75) in the Ctrl group and 13.79% (4/29) in the IA group (Figure 2I, Table 1). Statistical analysis demonstrated that 75% (9/12) of mice in the Ctrl group became pregnant, compared to 16.67% (4/24) in the IA group (Figure 2J, Table 2).

### 3.3. Systemic Levels of Inflammatory Cytokines in Pregnant Mice with IA

**Figure 3:**
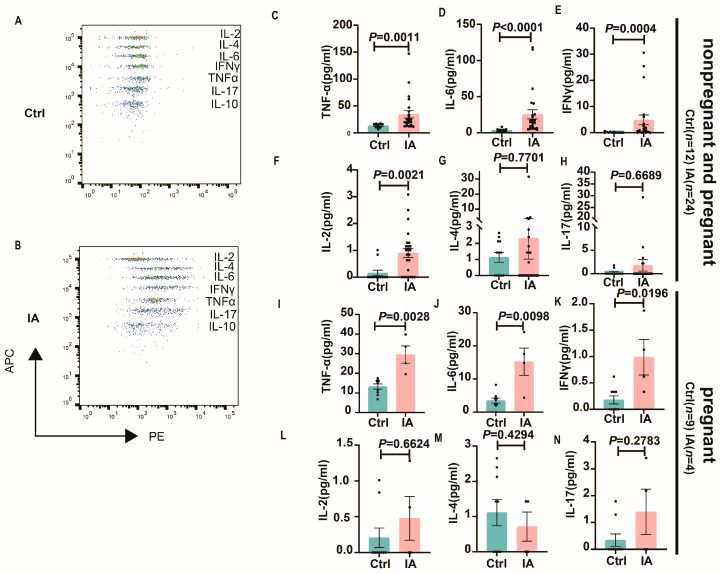
Plasma cytokine expression analysis in IA mice. (**A**) Representative flow cytometry plot of cytokine expression in a Ctrl group sample. (**B**) Representative flow cytometry plot of cytokine expression in an IA group sample. (**C**–**F**) Graphs illustrating the expression levels of Th1-related cytokines TNFα (**C**), IL-6 (**D**), IFNγ (**E**), and IL-2 (**F**) in the Ctrl and IA groups. (**G**) Graph showing the expression of Th2-related cytokine IL-4 in the Ctrl and IA groups. (**H**) Graph showing the expression of Th17-related cytokine IL-17 in the Ctrl and IA groups. (**I**–**L**) Graphs illustrating the expression levels of Th1-related cytokines TNFα (**I**), IL-6 (**J**), IFNγ (**K**), and IL-2 (**L**) in the pregnant Ctrl and IA groups. (**M**) Graph showing the expression of Th2-related cytokine IL-4 in the pregnant Ctrl and IA groups. (**N**) Graph showing the expression of Th17-related cytokine IL-17 in the pregnant Ctrl and IA groups.

To investigate the inflammatory microenvironment in IA pregnancy, the expression levels of cytokines associated with Th1, Th2, and Th17 were measured using a CBA kit. In both nonpregnant and pregnant mice, compared to the Ctrl group, IA mice exhibited significantly elevated plasma levels of TNFα (14.96 (10.72, 15.74) vs. 22.88 (20.41, 48.27), *p* = 0.0011), IL-6 (3.67 (2.21, 4.92) vs. 14.77 (12.23, 38.62), *p* < 0.0001), IFNγ (0.00 (0.00, 0.26) vs. 1.06 (0.98, 8.79), *p* = 0.0004), IL-2 (0.00 (0.00, 0.38) vs. 0.93 (0.55, 1.25), *p* = 0.0021), IL-4 (1.43 (0.45, 1.81) vs. 0.00 (0.00, 5.02), *p* = 0.7701) and IL-17 (0.00 (0.00, 0.71) vs. 0.00 (0.00, 4.33), *p* = 0.6689). In pregnant mice, compared to the Ctrl group, IA mice exhibited significantly elevated plasma levels of TNFα (14.96 (10.23, 16.27) vs. 29.35 (15.29, 43.74), *p* = 0.0028), IL-6 (3.67 (1.73, 5.27) vs. 16.2 (2.13, 28.33), *p* = 0.0098), IFNγ (0.00 (0.00, 0.36) vs. 0.86 (0.00, 2.06), *p* = 0.0196), IL-2 (0.00 (0.00, 0.52) vs. 0.32 (0.00, 1.45), *p* = 0.6624), IL-4 (1.43 (0.25, 1.98) vs. 0.72 (0.00, 2.03), *p* = 0.4294), and IL-17 (0.00 (0.00, 0.87) vs. 1.10 (0.00, 4.08), *p* = 0.2783) (Figure 3A–N).

### 3.4. Immune Subpopulations in the Spleen and Draining Lymph Nodes of Pregnant IA Mice

**Figure 4:**
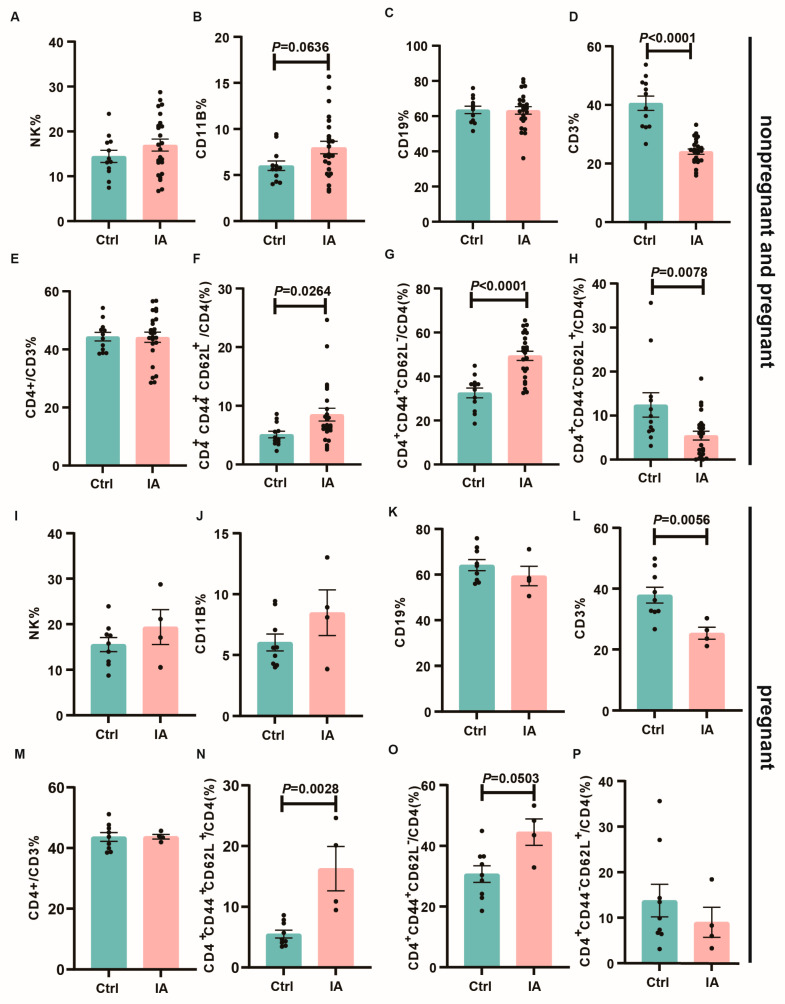
Immune cell subpopulations in the spleen. (**A**–**H**). Bar charts depicting the proportions of NK (**A**), CD11b (**B**), CD19 (**C**), CD3 (**D**), CD4 (**E**), Tcm (CD4^+^CD44^+^CD62L^+^, (**F**)), Tem (CD4^+^CD44^+^CD62L^−^, (**G)**), and Tn (CD4^+^CD44^−^CD62L^+^, (**H**)) cells in the spleen of Ctrl (*n* = 12) and IA (*n* = 24) mice. (**G**–**P**) Bar chart showing the proportion of NK (**I**), CD11b (**J**), CD19 (**K**), CD3 (**L**), CD4 (**M**), Tcm (CD4^+^CD44^+^CD62L^+^, (**N**)), Tem (CD4^+^CD44^+^CD62L^−^, (**O**)), and Tn (CD4^+^CD44^−^CD62L^+^, (**P**)) cells in the spleen of pregnant Ctrl (*n* = 9) and IA (*n* = 4) mice.

Flow cytometry was then utilized to identify immune cell populations within the spleens and mesenteric draining lymph nodes (DLNs). Compared to the Ctrl group, IA mice demonstrated increased frequencies of NK cells (13.57 (11.50, 17.37) vs. 16.51 (14.18, 19.72), *p* = 0.311) and CD11b+ myeloid cells (5.7 (4.88, 7.14) vs. 7.16 (6.58, 9.39), *p* = 0.0636), whereas CD3+T cells (41.34 (35.20, 45.99) vs. 24.26 (22.25, 25.91), *p* < 0.0001) exhibited decreased frequencies, with no significant alterations observed in B cells (64.30 (58.89, 68.18) vs. 64 (58.84, 67.61), *p* = 0.987) or CD4+ T cells (44.46 (41.11, 47.67) vs. 44.98 (40.54, 47.77), *p* = 0.753). Enhanced activation of both CD4+ T cells was observed, along with increases in effector memory (Tem, CD44^+^CD62L^−^) (34.93 (27.58, 37.50) vs. 51.26 (45.06, 53.76), *p* < 0.0001) and central memory (Tcm, CD44^+^CD62L^+^) T cells (4.48 (3.88, 6.34) vs. 6.67 (6.26, 10.75), *p* = 0.0264), whereas naïve (Tn, CD44^−^CD62L^+^) T cells (9.24 (6.31, 18.52) vs. 5.61 (3.41, 7.50), *p* = 0.0078) exhibited decreased levels (Figure 4A–H). This trend was similarly observed in the subgroup of successfully pregnant mice (Figure 4G–P).

**Figure 5:**
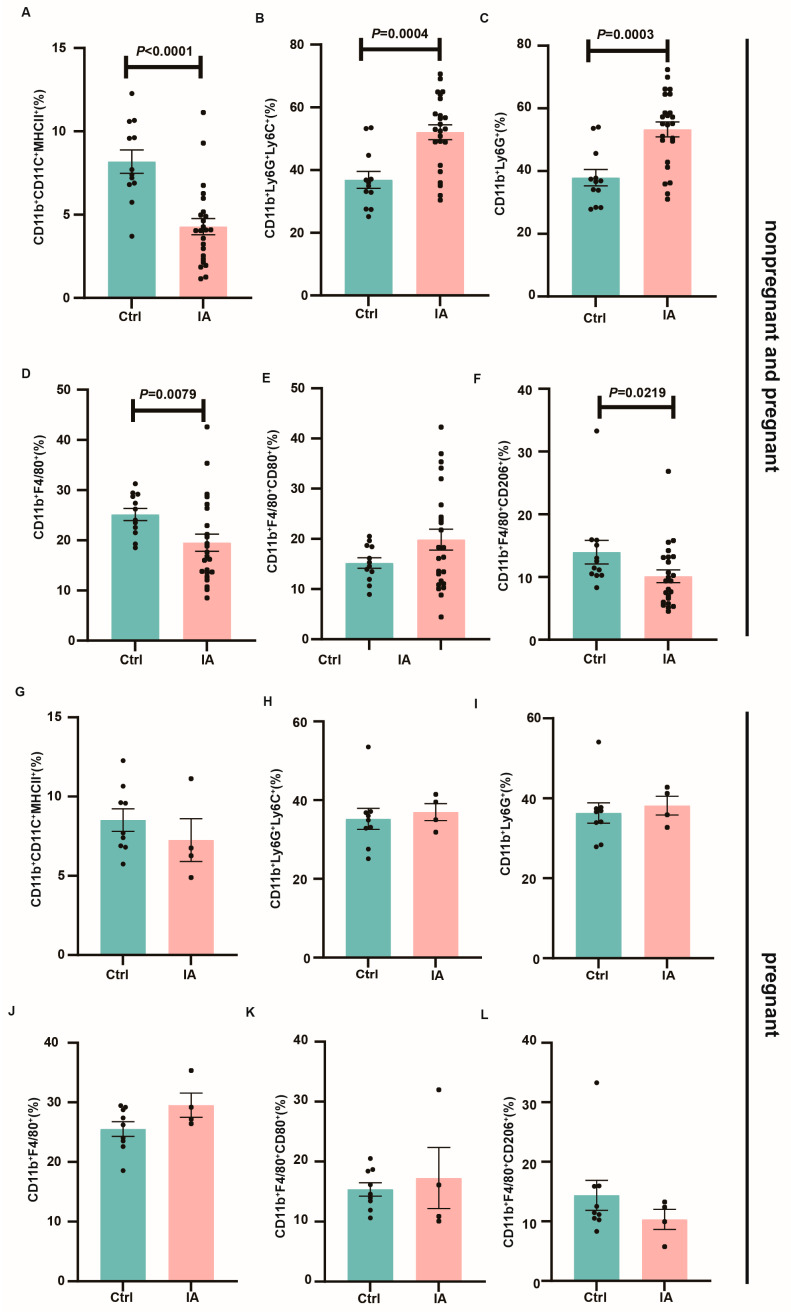
Myeloid immune cell subsets in the spleen and lymph nodes. (**A**–**F**). Bar charts showing the proportions of dendritic cells, monocytes, neutrophils, and macrophages in the spleen of Ctrl (*n* = 12) and IA (*n* = 24) mice. (**G**–**L**). Bar charts showing the proportions of dendritic cells, monocytes, neutrophils, and macrophages in the DLNs of pregnant Ctrl (*n* = 9) and IA (*n* = 4) mice.

Regarding myeloid immune cell subsets, IA mice exhibited higher proportions of monocytes (35.48 (30.96, 42.79) vs. 53.05 (47.22, 56.96), *p* = 0.0004), neutrophils (36.76 (32.20, 43.59) vs. 55.14 (48.35, 58.23), *p* = 0.0003), and M1 macrophages (14.95 (12.86, 17.51) vs. 17.31 (15.54, 24.19), *p* = 0.416) in both the spleen and DLNs, while dendritic cells (7.55 (6.65, 9.71) vs. 4.07 (3.27, 5.29), *p* < 0.0001) and M2 macrophages (11.98 (9.82, 18.13) vs. 9.38 (8.03, 12.23), *p* = 0.0219) were reduced (Figure 5A–F). Moreover, in the spleen and mesenteric DLNs of successfully pregnant mice, the trends in myeloid immune cell subset expression were similar between the Ctrl and IA groups, although the differences were not statistically significant (Figure 5G–L).

**Figure 6:**
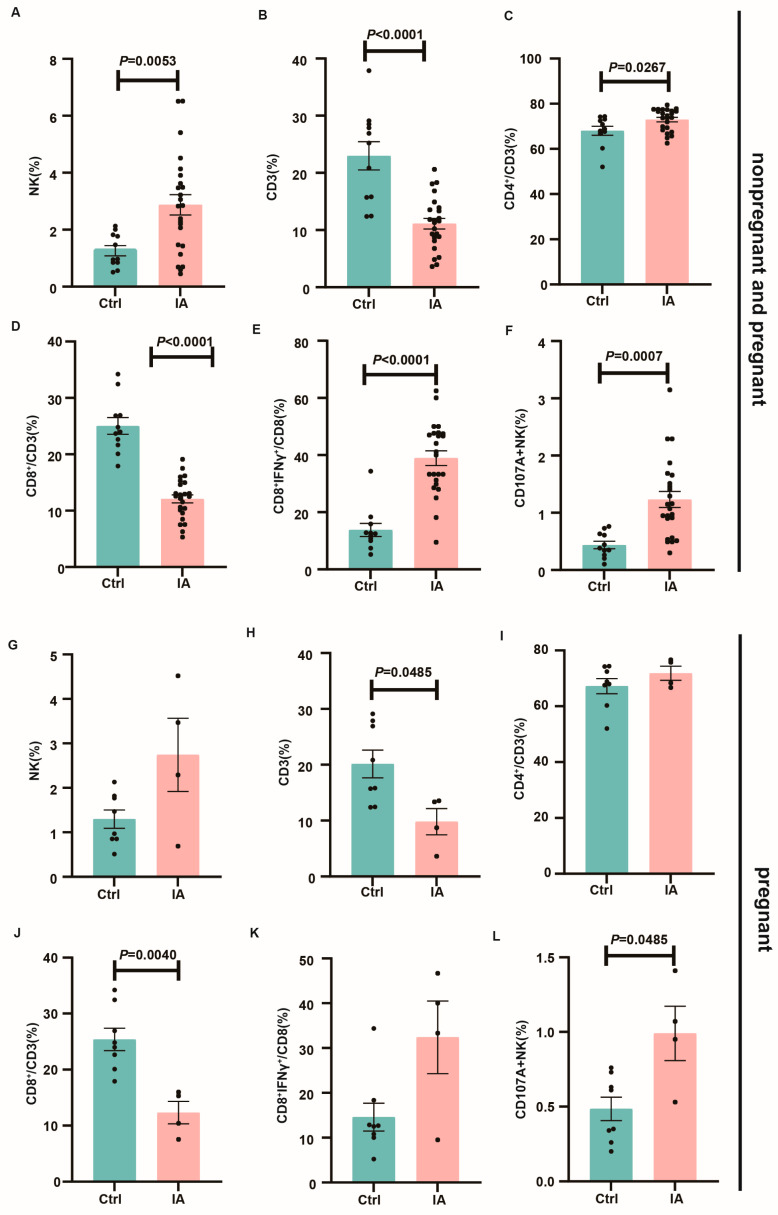
T cell and NK cell subpopulations in the spleen and lymph nodes. (**A**–**F**). Bar charts depicting the proportions of NK (**A**), CD3 (**B**), CD4 (**C**), CD8 (**D**), Tc1 (**E**), and CD107A+ NK cells (**F**) in the spleen of Ctrl (*n* = 12) and IA (*n* = 24) mice. (**G**–**L**). Bar charts depicting the proportions of NK (**G**), CD3 (**H**), CD4 (**I**), CD8 (**J**), Tc1 (**K**), and CD107A+ NK cells (**L**) in the spleen of pregnant Ctrl (*n* = 9) and IA (*n* = 4) mice.

Cytotoxic NK cells (CD107A+ NK) and T cell subpopulations, including Th1 (CD4+IFNγ+), Th17 (CD4+IL-17+), Tc1 (CD8+IFNγ+), and Tc17 (CD8+IL-17+) cells, were then analyzed. In contrast with the control, IA animals exhibited increased proportions of Tc1 (12.59 (9.25, 18.58) vs. 40.59 (33.61, 44.29), *p* < 0.0001) and CD107A+ NK cells (0.41 (0.30, 0.74) vs. 1.11 (0.00, 6.72), *p* = 0.0007) in both the spleen and DLNs (Figure 6E,F). Similarly, in the spleen as well as mesenteric DLNs of successfully pregnant mice, immune subpopulation patterns were consistent between the Ctrl and IA groups (Figure 6K–L).

### 3.5. Immune Microenvironment at the Maternal–Fetal Interface

**Figure 7:**
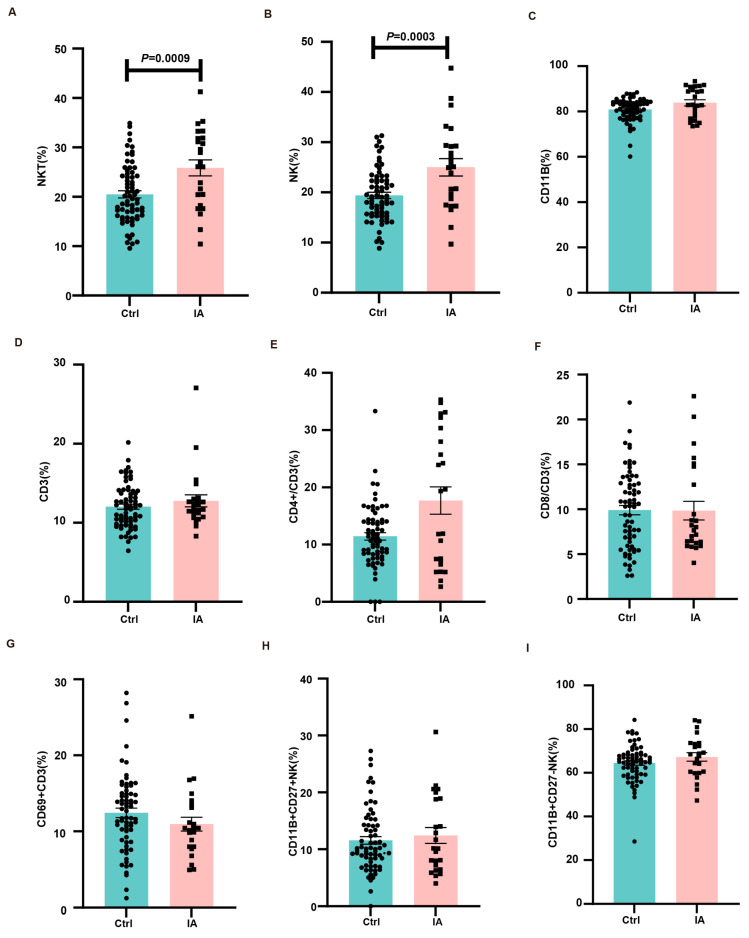
Immune cell subpopulations in the placenta. (**A**–**I**). Bar charts showing the proportions of NKT (**A**), NK (**B**), CD11b (**C**), CD3 (**D**), CD4 (**E**), CD8 (**F**), CD69+CD3 (**G**), CD11b^+^CD27^+^/NK (**H**), and CD11b^+^CD27^−^/NK (**I**) cells in the placenta of Ctrl (*n* = 68) and IA (*n* = 24) mice.

To examine alterations in maternal–fetal immune tolerance within the IA pregnancy microenvironment, single-cell suspensions from placental tissues were prepared, and immune cell segments were assessed through flow cytometry. Compared with the Ctrl group, IA pregnancies exhibited increased proportions of NKT (19.51 (19.04, 21.95) vs. 26.58 (22.53, 29.18), *p* = 0.0009) and NK (18.69 (18.14, 20.65) vs. 25.04 (21.42, 28.59), *p* = 0.0003) cells in the placenta (Figure 7A,B). In contrast, the frequencies of CD11b (82.06 (79.60, 82.07) vs. 82.95 (81.04, 86.68), *p* = 0.0907), CD3 (11.88 (11.37, 12.71) vs. 11.88 (11.18, 14.35), *p* = 0.5553), CD4 (10.91 (10.14, 12.75) vs. 15.63 (12.75, 22.63), *p* = 0.1564), CD8 (10.33 (8.87, 10.95) vs. 7.83 (7.69, 12.00), *p* = 0.7590), CD69+ CD3 (12.24 (11.19, 13.67) vs. 10.31 (9.06, 12.85), *p* = 0.0900), CD11b+CD27+/NK (10.11 (10.21, 12.90) vs. 10.14 (9.53, 15.30), *p* = 0.9331), and CD11b+CD27-/NK cells (65.05 (62.44, 66.48) vs. 68.04 (63.16, 71.26), *p* = 0.2010) remained unchanged between the groups (Figure 7C–I).

## 4. Discussion

Our study successfully established an IA pregnancy mouse model that mirrors several key aspects of the human disease, demonstrating impaired fertility and APOs. IA induction resulted in prolonged conception times, reduced successful mating, increased embryo resorption, and overall lower pregnancy rates compared to controls. These observations align with clinical findings that IA patients face challenges in conception and an elevated risk of complications such as intrauterine growth retardation, preterm birth, and reduced live birth rates [14,15,21,35].

Spiral artery remodeling is crucial for ensuring adequate nutrient and oxygen supply to the fetus. In humans, this process occurs in two stages: initially, spiral arterioles transform into high-capacity, low-resistance vessels independent of extravillous trophoblast (EVT) cells. The second stage involves EVT invasion and endothelial cell replacement by trophoblast cells [47]. Human decidual NK (dNK) cells promote this remodeling by secreting MMPs, Ang-1, Ang-2, and VEGF-α [48]. In mice, dNK cells also enhance angiogenesis and spiral artery remodeling, primarily through IFN-γ secretion [49]. The integrity of trophoblast cells is vital for placental function [50]. Chronic oxidative stress damages trophoblast cells, impairing placental structure and function [51]. Trophoblasts are particularly vulnerable to reactive oxygen species (ROS), which disrupt cell proliferation [52], apoptosis [53], and immune responses [54], negatively affecting placental angiogenesis. Key molecules like VEGF, NOS3, and IGF-1 are essential for normal placental function [55], and oxidative stress can impair angiogenesis, a critical factor shaping the placental barrier phenotype. The placenta is central to nutrient and waste exchange between mother and fetus, supporting fetal growth and development. Trophoblast cells facilitate nutrient transport and maintain tight junction integrity [56]. The placental barrier regulates essential nutrients, hormones, growth factors, and metabolic waste. Maternal nutrition, placental morphology, fetal-placental blood flow, and nutrient transport proteins all influence placental function and fetal development [57].

The clinical literature presents mixed findings regarding pregnancy outcomes in IA. Some studies have reported little distinction between patients with IA and healthy controls [58,59], whereas others, such as the study by Reed et al. [60], have identified an increased risk of premature delivery among patients with IA. The association between IA and PE remains controversial, with conflicting reports of elevated risk [25,32,61,62] versus no difference compared to the general population [63,64]. Our data support the notion that an imbalanced immune response, characterized by excessive pro-inflammatory cytokine production and altered immune cell populations, could be a driving factor behind these complications. Specifically, heightened inflammatory activity may impair placental angiogenesis and function, potentially via mechanisms such as reduced activity of 11β-hydroxysteroid dehydrogenase type 2 (11β-HSD2), leading to placental insufficiency and elevated maternal cortisol levels [65,66,67,68]. These factors have been implicated in adverse outcomes like preterm birth, LBW, and small-for-gestational-age infants (SGA) [69,70]. In this study, we used an animal model of IA pregnancy, demonstrating the impact of IA on pregnancy outcomes and the underlying immune mechanisms. We found that pregnant IA mice had prolonged conception times, reduced successful mating, increased embryo resorption, and lower pregnancy rates.

Pregnancy is inherently a state of active immune tolerance, necessitating precise adaptations to accept the semi-allogeneic fetus while maintaining effective pathogen defense [71]. Normally, shifts in lymphocyte subsets, including increased Treg cells and a balanced Th1/Th2 response, support fetal development [72,73,74,75]. However, in the IA context, these adaptations are disrupted. Our results show that IA induces an environment where pro-inflammatory signals predominate, undermining the typical immune modulation of pregnancy. This dysregulation is observed both peripherally and within the maternal–fetal milieu, potentially increasing the risk of complications through impaired placental development and function.

Peripheral innate immune alterations during pregnancy, such as increased monocyte and granulocyte counts [76,77,78,79], underscore the dynamic immune adaptations necessary for gestation. Cytokines, serving as critical regulators, orchestrate immune tolerance within the maternal–fetal environment, support angiogenesis, and facilitate tissue remodeling. In our study, IA mice exhibited elevated plasma levels of Th1 and Th17 cytokines, including TNFα, IFNγ, IL-6, IL-2, and IL-17, whereas Th2 cytokines, typically associated with healthy pregnancies, were relatively diminished [80]. Elevated IFNγ levels have been linked to several pregnancy complications [81], and inflammatory mediators like TNFα and IL-6 are known to inhibit placental angiogenesis, potentially leading to fetal growth restriction. Indeed, recent findings suggest that high maternal IL-6 levels correlate with LBW as well as SGA, while higher IL-10 levels are positively associated with fetal birth weight [82]. However, within the context of our model, no discernible difference in fetal weight was observed between the IA and control groups. This is probably because we only monitored the fetal weight at P15, a time point at which the fetus had not entered the fast-growing phase.

The mechanisms driving immune adaptation during pregnancy remain incompletely defined. While semi-allogeneic fetal tissue partially accounts for maternal immune modulation, similar alterations have been observed in syngeneic pregnancy models [83]. Hormonal shifts, particularly increased progesterone levels, are also implicated in these adaptations [84]. Additionally, direct placental contact during circulation and the release of various placental factors, including cytokines, extracellular vesicles, and fetal DNA, further influence maternal immune responses [85,86,87]. In the context of IA, where multiple immune cell types such as T cells, B cells, neutrophils, and macrophages are involved, the balance between effective pathogen defense and fetal tolerance is particularly critical.

Our IA mouse model revealed significant alterations in the populations of immune cells. The proportions of NK and CD11b+ myeloid cells were increased in both the spleen and DLNs, whereas CD3+ T cells were decreased. Enhanced activation of CD4+ T cells was observed, along with increases in Tem and Tcm cells. Moreover, the proportions of monocytes, neutrophils, and M1 macrophages were elevated, whereas dendritic cells and M2 macrophages were reduced. Conversely, the rising frequencies of Tc1 and NK+CD107A cells indicated a similar transition toward pro-inflammatory activity. The placenta of IA mice exhibited heightened infiltration of NKT, NK, CD11b, and CD4+ T cells. Under normal pregnancy conditions, NK cell cytotoxicity and IFNγ secretion are downregulated to protect placental integrity [88]. However, the elevated presence of NK cells in IA may lead to cytotoxic activity within the decidua, potentially disrupting trophoblast function and angiogenesis through the secretion of IFNγ and TNFα [89,90,91].

dNK cells—distinct from their peripheral counterparts—represent the primary immune cells within the placenta [92]. They facilitate placental vascular remodeling by secreting factors such as VEGF, PLGF, and Angiopoietin-2 [48], and they modulate immune responses through interactions with trophoblast HLA molecules via killer cell immunoglobulin-like receptors (KIRs) [93]. Furthermore, by secreting IL-10 and TGF-β [90], dNK cells inhibit maternal T cell activation and promote trophoblast invasion through the release of matrix metalloproteinases (MMP-2 and MMP-9) [94].

Macrophages, as versatile components of the innate immune system, play central roles in inflammation, host defense, and tissue homeostasis. Their phenotype and function are highly plastic, adapting to local microenvironmental cues. Within the placenta, macrophages contribute to tissue repair, immune tolerance, and angiogenesis [95,96]. However, in IA, an imbalance toward a pro-inflammatory state is evident, with increased M1 macrophages and decreased M2 macrophages [97]. This shift may exacerbate placental ischemia and endothelial damage, triggering inflammatory cascades that impair the clearance of apoptotic cells, potentially leading to fibrosis within the villous space [98].

T cell dynamics during pregnancy are crucial for balancing maternal immune defense and fetal tolerance. Normally, an increase in regulatory T cells (Tregs) and a Th2-skewed response help mitigate inflammatory damage. Cytokines secreted by T helper (Th) cells play a crucial role in maintaining immune tolerance at the maternal–fetal interface. The Th1/Th2 cell balance is vital for maternal–fetal tolerance: Th1 promotes inflammation and tissue damage, hindering embryo implantation and trophoblast cell function, while Th2 supports immune suppression, promotes embryo implantation, and facilitates fetal development. A Shift in the Th1/Th2 balance towards Th2 dominance and temporary inactivation of Th1 cells is essential for fetal survival during pregnancy [99]. In contrast, our IA model exhibited an increased proportion of Tc1 cells at the maternal–fetal interface, suggesting a skew toward a pro-inflammatory state. DCs, as key antigen-presenting cells, also undergo dynamic changes during gestation to promote immune tolerance within the maternal–fetal environment. Their specific role in IA-related immune dysregulation during pregnancy remains inadequately investigated, even though their peripheral numbers tend to decline, potentially due to migration to the decidua.

The immune microenvironment at the maternal–fetal interface consists of decidual immune cells, stromal cells, and embryonic trophoblast cells. Early in pregnancy, immune cells make up a substantial portion of the decidual tissue, with NK, macrophages, and T cells being the major populations. Dendritic cells, B cells, and NKTs are present in small proportions [92]. dNK cells promote trophoblast invasion, vascular remodeling, and the maintenance of immune tolerance [100]. Before embryo implantation, decidual macrophages primarily exhibit the M1 phenotype. Upon trophoblast invasion, macrophages polarize into a mixed M1/M2 population, with M2 macrophages dominating after placental development and throughout pregnancy [101]. The M1/M2 balance is crucial for embryo implantation, maternal–fetal immune tolerance, spiral artery remodeling, and labor initiation. M2 macrophages promote the differentiation of dNK cells into a less cytotoxic, cytokine-secreting subset, thereby maintaining immune tolerance [102]. Additionally, macrophages produce proteolytic enzymes (e.g., MMP-7, MMP-9) that facilitate spiral artery remodeling and trophoblast invasion [103]. In late pregnancy, macrophages increase the production of inflammatory mediators, such as IL-1β, IL-6, TNF-α, and MMPs [104], contributing to labor initiation. Thus, maintaining a dynamic balance between M1 and M2 macrophages is essential for a successful pregnancy. At the maternal–fetal interface, CD4+ and CD8+ T cells play vital roles [105]. CD4+ T cells differentiate into Th1, Th2, Th17, and regulatory T cells [106]. The balance between Th1/Th2 and Th17/Treg cells shifts towards Th2 and Tregs during pregnancy. Disruption of these immune balances can lead to pregnancy complications [107]. In future work, we plan to investigate these mechanistic links through molecular analyses such as assessment of angiogenic factors, such as VEGF, or markers of trophoblast invasion, such as MMP9, which will provide more direct evidence of placental dysfunction.

Various immune cells, including CD4+ T cells, macrophages, neutrophils, and B cells, contribute to the pathogenesis of IA [108]. The imbalance of Th17/Treg and Th1/Th2 cells is strongly implicated in disease progression, with Th1 and Th17 cells producing pro-inflammatory cytokines and Th2 and Treg cells exerting anti-inflammatory effects [109]. Macrophages, particularly in the M1 phenotype, secrete cytokines such as TNF-α, IL-1β, and IL-6, driving inflammation [110]. Additionally, activated neutrophils and B cells release enzymes and autoantibodies, contributing to cartilage degradation and tissue damage [111]. Furthermore, macrophages play a pivotal role in regulating immune responses and can polarize between M1 and M2 phenotypes, depending on the stimuli. This polarization affects their pro-inflammatory or anti-inflammatory roles in IA progression. Recent studies also highlight the importance of innate immunity, with neutrophils and macrophages releasing reactive species and extracellular traps that exacerbate disease progression. These findings strengthen our understanding of the immune mechanisms underlying IA and may inform potential therapeutic strategies targeting immune modulation. In RSA, studies show that dNK cells exhibit higher cytotoxicity, with altered expression of CD49a, perforin, granzyme B, and IFN-γ compared to healthy controls [112]. Additionally, the Th17/Treg imbalance and elevated IL-23 in RSA patients contribute to immune dysregulation [113]. Similarly, an M1/M2 macrophage imbalance, with M1 dominance, has been linked to impaired trophoblast invasion and RSA pathogenesis [114].

## 5. Conclusions

In summary, the current research employed an IA pregnancy mouse model that effectively recapitulates the complex immune dysregulation observed in patients, demonstrating impaired fertility, prolonged conception times, and reduced pregnancy success rates. The pro-inflammatory immune microenvironment, characterized by altered cytokine profiles and immune cell distributions, appears to be a critical factor driving APOs. Further research is warranted to dissect the precise mechanisms by which these immune alterations influence placental function and pregnancy outcomes in IA, which may inform the development of targeted therapeutic strategies.

## 6. Strengths and Limitations

The strength of this study lies in its use of the SKG mouse model, which recapitulates human RA immunopathology and allows longitudinal tracking of pregnancy outcomes. Combined multi-tissue immune profiling (spleen, lymph nodes, placenta) strengthens the systemic relevance of findings. However, limitations include the following: (1) Sample size: One limitation of this study is the relatively small sample size. Future studies with an expanded sample size are warranted to validate the robustness of the results. The single-strain mouse model may not fully capture human RA heterogeneity. (2) Translational gaps: While mechanistic pathways are identified, therapeutic validation (e.g., cytokine blockade) remains to be conducted.

## 7. Interpretation

The observed Th1/Th17/Tc1 skewing suggests that RA disrupts maternal–fetal tolerance via excessive cytotoxic immunity, potentially through activated antigen-presenting cells priming T-cell responses. Placental infiltration of NKT/NK cells may directly damage trophoblasts, while M2 macrophage depletion compromises tissue repair. These mechanisms mirror autoimmune-driven pregnancy complications (e.g., lupus), supporting a shared pathway of immune-mediated APOs. Clinically, this underscores the need for pre-conception immunomodulation in RA patients. Future studies should target specific cytokines (e.g., IFN-γ, TNF-α, and IL-17A) to test the reversibility of APOs in this model.

## Figures and Tables

**Table 1:** Embryo resorption rate.

Group	Number (*n*)	Total Number of Embryos (*n*)	Number of Absorbed Embryos (*n*)	Rate of Embryo Absorption (%)
Ctrl	12	75	4	5.33
IA	24	29	4	13.79

**Table 2:** Pregnancy rate.

Group	Number (*n*)	With Vaginal Plug (*n*)	Without Vaginal Plug (*n*)	Successful Pregnancy (*n*)	Pregnancy Failure (*n*)	Pregnancy Rate (%)
Ctrl	12	12	0	9	3	75
IA	24	13	11	4	20	16.67

## Data Availability

The original contributions presented in the study are included in the article, further inquiries can be directed to the corresponding author.

## References

[1] Perretti M., Cooper D., Dalli J., Norling L.V. (2017). Immune resolution mechanisms in inflammatory arthritis. Nat. Rev. Rheumatol..

[2] Wu X. (2020). Innate Lymphocytes in Inflammatory Arthritis. Front. Immunol..

[3] Iwaszko M., Biały S., Bogunia-Kubik K. (2021). Significance of Interleukin (IL)-4 and IL-13 in Inflammatory Arthritis. Cells.

[4] Badley E.M., Kasman N.M. (2004). The Impact of Arthritis on Canadian Women. BMC Womens Health.

[5] Märker-Hermann E., Bauer H., Gromnica-Ihle E. (2008). Rheumatic diseases in pregnancy. Dtsch. Med. Wochenschr..

[6] Mehta B., Luo Y., Xu J., Sammaritano L., Salmon J., Lockshin M., Goodman S., Ibrahim S. (2019). Trends in Maternal and Fetal Outcomes Among Pregnant Women with Systemic Lupus Erythematosus in the United States: A Cross-sectional Analysis. Ann. Intern. Med..

[7] Smeele H.T.W., Dolhain R. (2019). Current perspectives on fertility, pregnancy and childbirth in patients with Rheumatoid Arthritis. Semin. Arthritis Rheum..

[8] Park E.H., Lee J.S., Kim Y.J., Lee S.M., Jun J.K., Lee E.B., Kim Y.G. (2019). Pregnancy outcomes in Korean women with ankylosing spondylitis. Korean J. Intern. Med..

[9] Chen Y.J., Chang J.C., Lai E.L., Liao T.L., Chen H.H., Hung W.T., Hsieh T.Y., Huang W.N., Chen Y.H., Lin C.H. (2020). Maternal and perinatal outcomes of pregnancies in systemic lupus erythematosus: A nationwide population-based study. Semin. Arthritis Rheum..

[10] Jakobsson G., Stephansson O., Askling J., Jacobsson L. (2016). Pregnancy outcomes in patients with ankylosing spondylitis: A nationwide register study. Ann. Rheum. Dis..

[11] Unal C., Fadiloglu E., Tanacan A., Zaim O., Beksac M. (2020). Retrospective evaluation of pregnancies with ankylosing spondylitis in a tertiary center in Turkey. Int. J. Rheum. Dis..

[12] Kishore S., Mittal V., Majithia V. (2019). Obstetric outcomes in women with rheumatoid arthritis: Results from Nationwide Inpatient Sample Database 2003–2011. Semin. Arthritis Rheum..

[13] Zbinden A., van den Brandt S., Østensen M., Villiger P., Förger F. (2018). Risk for adverse pregnancy outcome in axial spondyloarthritis and rheumatoid arthritis: Disease activity matters. Rheumatology.

[14] Nørgård B., Larsen M., Friedman S., Knudsen T., Fedder J. (2019). Decreased chance of a live born child in women with rheumatoid arthritis after assisted reproduction treatment: A nationwide cohort study. Ann. Rheum. Dis..

[15] Vinet E., Labrecque J., Pineau C., Clarke A., St-Pierre Y., Platt R., Bernatsky S. (2012). A population-based assessment of live births in women with systemic lupus erythematosus. Ann. Rheum. Dis..

[16] Rudwaleit M., Landewé R., van der Heijde D., Listing J., Brandt J., Braun J., Burgos-Vargas R., Collantes-Estevez E., Davis J., Dijkmans B. (2009). The development of Assessment of SpondyloArthritis international Society classification criteria for axial spondyloarthritis (part I): Classification of paper patients by expert opinion including uncertainty appraisal. Ann. Rheum. Dis..

[17] Lavender T., Hofmeyr G.J., Neilson J.P., Kingdon C., Gyte G.M. (2012). Caesarean section for non-medical reasons at term. Cochrane Database Syst. Rev..

[18] Mørk S., Voss A., Möller S., Bliddal M. (2019). Spondyloarthritis and Outcomes in Pregnancy and Labor: A Nationwide Register-Based Cohort Study. Arthritis Care Res..

[19] Maguire S., Molto A. (2023). Pregnancy & neonatal outcomes in spondyloarthritis. Best Pract. Res. Clin. Rheumatol..

[20] Zucchi D., Tani C., Mosca M. (2024). Reproductive Health in RA, Lupus, and APS. J. Clin. Rheumatol..

[21] Nakai T., Fukui S., Ozawa H., Kitada A., Okada M., Kishimoto M. (2024). Management of pregnant with rheumatoid arthritis: Preconception care, pregnancy and lactation strategies, and maternal-fetal outcomes. Best Pract. Res. Clin. Rheumatol..

[22] Förger F., Villiger P. (2020). Immunological adaptations in pregnancy that modulate rheumatoid arthritis disease activity. Nat. Rev. Rheumatol..

[23] Pina Vegas L., Drouin J., Weill A., Dray-Spira R. (2024). Pregnancy outcomes in women with rheumatoid arthritis: An 11-year French nationwide study. RMD Open.

[24] Tsai Y., Chang H., Chiou M., Luo S., Kuo C. (2022). Fetal-neonatal and maternal pregnancy outcomes in women with rheumatoid arthritis: A population-based cohort study. BMJ Open.

[25] Nørgaard M., Larsson H., Pedersen L., Granath F., Askling J., Kieler H., Ekbom A., Sørensen H., Stephansson O. (2010). Rheumatoid arthritis and birth outcomes: A Danish and Swedish nationwide prevalence study. J. Intern. Med..

[26] Strouse J., Donovan B., Fatima M., Fernandez-Ruiz R., Baer R., Nidey N., Forbess C., Bandoli G., Paynter R., Parikh N. (2019). Impact of autoimmune rheumatic diseases on birth outcomes: A population-based study. RMD Open.

[27] Bharti B., Lee S., Lindsay S., Wingard D., Jones K., Lemus H., Chambers C. (2015). Disease Severity and Pregnancy Outcomes in Women with Rheumatoid Arthritis: Results from the Organization of Teratology Information Specialists Autoimmune Diseases in Pregnancy Project. J. Rheumatol..

[28] de Steenwinkel F., Hokken-Koelega A., de Man Y., de Rijke Y., de Ridder M., Hazes J., Dolhain R. (2013). Circulating maternal cytokines influence fetal growth in pregnant women with rheumatoid arthritis. Ann. Rheum. Dis..

[29] de Jong P., Dolhain R. (2017). Fertility, Pregnancy, and Lactation in Rheumatoid Arthritis. Rheum. Dis. Clin. N. Am..

[30] Pósfai É., Bánhidy F., Urbán R., Czeizel A. (2015). Birth Outcomes of Children Born to Women with Rheumatoid Arthritis. Cent. Eur. J. Public Health.

[31] Jølving L., Nielsen J., Kesmodel U., Nielsen R., Beck-Nielsen S., Nørgård B. (2018). Children Born by Women with Rheumatoid Arthritis and Increased Susceptibility for Chronic Diseases: A Nationwide Cohort Study. Arthritis Care Res..

[32] Huang W., Wu T., Jin T., Zhang Y., Wang J., Qi J., Li Y., Jiang H., Zhang J., Jiang Z. (2023). Maternal and fetal outcomes in pregnant women with rheumatoid arthritis: A systematic review and meta-analysis. Clin. Rheumatol..

[33] Al Rayes H., Abdulaziz S., Alotaibi A., Alaithan M., Attar M., Daghasi H., Melibari R., Althagafi A., Elnady B. (2021). Adverse Impact of Rheumatoid Arthritis on Pregnancy Outcomes: A Saudi Arabia Prospective Multicenter Study. Open Access Rheumatol..

[34] Smith C., Förger F., Bandoli G., Chambers C. (2019). Factors Associated with Preterm Delivery Among Women with Rheumatoid Arthritis and Women with Juvenile Idiopathic Arthritis. Arthritis Care Res..

[35] Lv J., Xu L., Mao S. (2023). Association between disease activity of rheumatoid arthritis and maternal and fetal outcomes in pregnant women: A systematic review and meta-analysis. BMC Pregnancy Childbirth.

[36] Chen J., Roberts C., Simpson J., March L. (2015). Pregnancy Outcomes in Women with Rare Autoimmune Diseases. Arthritis Rheumatol..

[37] Ostensen M., Brucato A., Carp H., Chambers C., Dolhain R., Doria A., Förger F., Gordon C., Hahn S., Khamashta M. (2011). Pregnancy and reproduction in autoimmune rheumatic diseases. Rheumatology.

[38] Sammaritano L., Bermas B., Chakravarty E., Chambers C., Clowse M., Lockshin M., Marder W., Guyatt G., Branch D., Buyon J. (2020). 2020 American College of Rheumatology Guideline for the Management of Reproductive Health in Rheumatic and Musculoskeletal Diseases. Arthritis Rheumatol..

[39] Sakaguchi N., Takahashi T., Hata H., Nomura T., Tagami T., Yamazaki S., Sakihama T., Matsutani T., Negishi I., Nakatsuru S. (2003). Altered thymic T-cell selection due to a mutation of the ZAP-70 gene causes autoimmune arthritis in mice. Nature.

[40] Boissier M.C., Bessis N. (2017). Do we need animal models to advance research on inflammatory joint disease?. Jt. Bone Spine.

[41] Nakamura A., Jo S., Nakamura S., Aparnathi M.K., Boroojeni S.F., Korshko M., Park Y.S., Gupta H., Vijayan S., Rockel J.S. (2024). HIF-1α and MIF enhance neutrophil-driven type 3 immunity and chondrogenesis in a murine spondyloarthritis model. Cell. Mol. Immunol..

[42] Jeong H., Bae E.K., Kim H., Lim D.H., Chung T.Y., Lee J., Jeon C.H., Koh E.M., Cha H.S. (2017). Spondyloarthritis features in zymosan-induced SKG mice. Jt. Bone Spine.

[43] Ruutu M., Thomas G., Steck R., Degli-Esposti M.A., Zinkernagel M.S., Alexander K., Velasco J., Strutton G., Tran A., Benham H. (2012). β-glucan triggers spondylarthritis and Crohn’s disease-like ileitis in SKG mice. Arthritis Rheumatol..

[44] Rahman M.A., Thomas R. (2018). The SKG model of spondyloarthritis. Best Pract. Res. Clin. Rheumatol..

[45] Keller K., Lindgaard L., Wogensen L., Dagnæs-Hansen F., Thomsen J., Sakaguchi S., Stengaard-Pedersen K., Hauge E. (2013). SKG arthritis as a model for evaluating therapies in rheumatoid arthritis with special focus on bone changes. Rheumatol. Int..

[46] Gao Q.W., Liu W.Y., Jawad M., Ci L., Cao Y.Y., Xi J., Wu J.Y., Lei Y.Y., Hu Y.S., You X.Y. (2025). Aristolochic acid IVa ameliorates arthritis in SKG Mice by regulating macrophage polarization and Th17/Treg balance. Phytomedicine.

[47] Erlebacher A. (2013). Immunology of the maternal-fetal interface. Annu. Rev. Immunol..

[48] Kalkunte S.S., Mselle T.F., Norris W.E., Wira C.R., Sentman C.L., Sharma S. (2009). Vascular endothelial growth factor C facilitates immune tolerance and endovascular activity of human uterine NK cells at the maternal-fetal interface. J. Immunol..

[49] Ashkar A., Di Santo J., Croy B. (2000). Interferon gamma contributes to initiation of uterine vascular modification, decidual integrity, and uterine natural killer cell maturation during normal murine pregnancy. J. Exp. Med..

[50] Shi L., Wang Z., Xiao J., Hu R., Zou H., Wang J., Yue Z., Peng Q., Jiang Y., Xue B. (2025). Folic Acid Alleviates Hydrogen Peroxide-Induced Oxidative Stress in Bovine Placental Trophoblast Cells by Regulating the NRF2/mTOR Signaling Pathway. Int. J. Mol. Sci..

[51] Ye L., Huang Y., Liu X., Zhang X., Cao Y., Kong X., Yuan X., Xu J., Zhu H. (2023). Apelin/APJ system protects placental trophoblasts from hypoxia-induced oxidative stress through activating PI3K/Akt signaling pathway in preeclampsia. Free Radic. Biol. Med..

[52] Kilburn B., Wang J., Duniec-Dmuchowski Z., Leach R., Romero R., Armant D. (2000). Extracellular matrix composition and hypoxia regulate the expression of HLA-G and integrins in a human trophoblast cell line. Biol. Reprod..

[53] Pirković A., Vilotić A., Borozan S., Nacka-Aleksić M., Bojić-Trbojević Ž., Krivokuća M., Battino M., Giampieri F., Dekanski D. (2023). Oleuropein Attenuates Oxidative Stress in Human Trophoblast Cells. Antioxidants.

[54] Wu F., Tian F., Lin Y., Xu W. (2016). Oxidative Stress: Placenta Function and Dysfunction. Am. J. Reprod. Immunol..

[55] Barut A., Barut F., Kandemir N., Aktunc E., Arikan I., Harma M., Harma M., Gun B. (2012). Placental chorangiosis: The association with oxidative stress and angiogenesis. Gynecol. Obstet. Investig..

[56] Karahoda R., Zaugg J., Fuenzalida B., Kallol S., Moser-Haessig R., Staud F., Albrecht C. (2022). Trophoblast Differentiation Affects Crucial Nutritive Functions of Placental Membrane Transporters. Front. Cell Dev. Biol..

[57] Cui C., Wu C., Wang J., Zheng X., Ma Z., Zhu P., Guan W., Zhang S., Chen F. (2022). Leucine supplementation during late gestation globally alters placental metabolism and nutrient transport via modulation of the PI3K/AKT/mTOR signaling pathway in sows. Food Funct..

[58] Brouwer J., Laven J.S., Hazes J.M., Dolhain R.J. (2015). Brief Report: Miscarriages in Female Rheumatoid Arthritis Patients: Associations with Serologic Findings, Disease Activity, and Antirheumatic Drug Treatment. Arthritis Rheumatol..

[59] Eudy A.M., McDaniel G., Clowse M.E. (2017). Pregnancy in rheumatoid arthritis: A retrospective study. Clin. Rheumatol..

[60] Reed S.D., Vollan T.A., Svec M.A. (2006). Pregnancy outcomes in women with rheumatoid arthritis in Washington State. Matern. Child Health J..

[61] Aljary H., Czuzoj-Shulman N., Spence A.R., Abenhaim H.A. (2018). Pregnancy outcomes in women with rheumatoid arthritis: A retrospective population-based cohort study. J. Matern. Fetal Neonatal Med..

[62] Jacobsson L.T., Jacobsson M.E., Askling J., Knowler W.C. (2003). Perinatal characteristics and risk of rheumatoid arthritis. Br. Med. J..

[63] Williams A., Grantz K., Seeni I., Robledo C., Li S., Ouidir M., Nobles C., Mendola P. (2019). Obstetric and neonatal complications among women with autoimmune disease. J. Autoimmun..

[64] Wallenius M., Salvesen K.Å., Daltveit A.K., Skomsvoll J.F. (2013). Rheumatoid arthritis and outcomes in first and subsequent births based on data from a national birth registry. Acta Obstet. Gynecol. Scand..

[65] Wolfberg A.J., Lee-Parritz A., Peller A.J., Lieberman E.S. (2004). Association of rheumatologic disease with preeclampsia. Obstet. Gynecol..

[66] Harmon A.C., Cornelius D.C., Amaral L.M., Faulkner J.L., Cunningham M.W., Wallace K., LaMarca B. (2016). The role of inflammation in the pathology of preeclampsia. Clin. Sci..

[67] Motta F., Codullo V., Ramoni V., Cesari S., Ferrario G., Fiandrino G., Beneventi F., Rampello S., Johnsson H., Montecucco C. (2020). Role of placental inflammatory mediators and growth factors in patients with rheumatic diseases with a focus on systemic sclerosis. Rheumatology.

[68] Kossintseva I., Wong S., Johnstone E., Guilbert L., Olson D.M., Mitchell B.F. (2005). Proinflammatory cytokines inhibit human placental 11beta-hydroxysteroid dehydrogenase type 2 activity through Ca^2+^ and cAMP pathways. Am. J. Physiol. Endocrinol. Metab..

[69] Chen Z., Bozec A., Ramming A., Schett G. (2018). Anti-inflammatory and immune-regulatory cytokines in rheumatoid arthritis. Nat. Rev. Rheumatol..

[70] Fowden A.L., Valenzuela O.A., Vaughan O.R., Jellyman J.K., Forhead A.J. (2016). Glucocorticoid programming of intrauterine development. Domest. Anim. Endocrinol..

[71] Veenstra van Nieuwenhoven A.L., Heineman M.J., Faas M.M. (2003). The immunology of successful pregnancy. Hum. Reprod. Update.

[72] Faas M.M., de Vos P. (2017). Uterine NK cells and macrophages in pregnancy. Placenta.

[73] Saito S., Tsukaguchi N., Hasegawa T., Michimata T., Tsuda H., Narita N. (1999). Distribution of Th1, Th2, and Th0 and the Th1/Th2 cell ratios in human peripheral and endometrial T cells. Am. J. Reprod. Immunol..

[74] Santner-Nanan B., Peek M.J., Khanam R., Richarts L., Zhu E., Fazekas de St Groth B., Nanan R. (2009). Systemic increase in the ratio between Foxp3+ and IL-17-producing CD4+ T cells in healthy pregnancy but not in preeclampsia. J. Immunol..

[75] Saito S., Nakashima A., Shima T., Ito M. (2010). Th1/Th2/Th17 and regulatory T-cell paradigm in pregnancy. Am. J. Reprod. Immunol..

[76] Veenstra van Nieuwenhoven A.L., Bouman A., Moes H., Heineman M.J., de Leij L.F., Santema J., Faas M.M. (2003). Endotoxin-induced cytokine production of monocytes of third-trimester pregnant women compared with women in the follicular phase of the menstrual cycle. Am. J. Obstet. Gynecol..

[77] Luppi P., Deloia J.A. (2006). Monocytes of preeclamptic women spontaneously synthesize pro-inflammatory cytokines. Clin. Immunol..

[78] Faas M.M., Kunnen A., Dekker D.C., Harmsen H.J., Aarnoudse J.G., Abbas F., De Vos P., Van Pampus M.G. (2014). Porphyromonas Gingivalis and E-coli induce different cytokine production patterns in pregnant women. PLoS ONE.

[79] Melgert B.N., Spaans F., Borghuis T., Klok P.A., Groen B., Bolt A., de Vos P., van Pampus M.G., Wong T.Y., van Goor H. (2012). Pregnancy and preeclampsia affect monocyte subsets in humans and rats. PLoS ONE.

[80] Piccinni M., Lombardelli L., Logiodice F., Kullolli O., Romagnani S., Le Bouteiller P. (2015). T helper cell mediated-tolerance towards fetal allograft in successful pregnancy. Clin. Mol. Allergy.

[81] Nurzadeh M., Ghalandarpoor-Attar S., Ghalandarpoor-Attar S., Rabiei M. (2023). The Role of Interferon (IFN)-γ in Extravillous Trophoblast Cell (EVT) Invasion and Preeclampsia Progression. Reprod. Sci..

[82] Atta D.S., Girbash E.F., Abdelwahab S.M., Abdeldayem H.M., Tharwat I., Ghonaim R. (2015). Maternal cytokines and disease severity influence pregnancy outcomes in women with rheumatoid arthritis. J. Matern. Fetal Neonatal Med..

[83] Elderman M., Hugenholtz F., Belzer C., Boekschoten M., de Haan B., de Vos P., Faas M. (2018). Changes in intestinal gene expression and microbiota composition during late pregnancy are mouse strain dependent. Sci. Rep..

[84] Szekeres-Bartho J., Barakonyi A., Par G., Polgar B., Palkovics T., Szereday L. (2001). Progesterone as an immunomodulatory molecule. Int. Immunopharmacol..

[85] Mellembakken J.R., Aukrust P., Olafsen M.K., Ueland T., Hestdal K., Videm V. (2002). Activation of leukocytes during the uteroplacental passage in preeclampsia. Hypertension.

[86] Sacks G.P., Clover L.M., Bainbridge D.R., Redman C.W., Sargent I.L. (2001). Flow cytometric measurement of intracellular Th1 and Th2 cytokine production by human villous and extravillous cytotrophoblast. Placenta.

[87] Göhner C., Plösch T., Faas M.M. (2017). Immune-modulatory effects of syncytiotrophoblast extracellular vesicles in pregnancy and preeclampsia. Placenta.

[88] Vacca P., Vitale C., Montaldo E., Conte R., Cantoni C., Fulcheri E., Darretta V., Moretta L., Mingari M.C. (2011). CD34+ hematopoietic precursors are present in human decidua and differentiate into natural killer cells upon interaction with stromal cells. Proc. Natl. Acad. Sci. USA.

[89] Fu B., Li X., Sun R., Tong X., Ling B., Tian Z., Wei H. (2012). Natural killer cells promote immune tolerance by regulating inflammatory TH17 cells at the human maternal-fetal interface. Proc. Natl. Acad. Sci. USA.

[90] Li X.F., Charnock-Jones D.S., Zhang E., Hiby S., Malik S., Day K., Licence D., Bowen J.M., Gardner L., King A. (2001). Angiogenic growth factor messenger ribonucleic acids in uterine natural killer cells. J. Clin. Endocrinol. Metab..

[91] Fu B., Zhou Y., Ni X., Tong X., Xu X., Dong Z., Sun R., Tian Z., Wei H. (2017). Natural Killer Cells Promote Fetal Development through the Secretion of Growth-Promoting Factors. Immunity.

[92] Zhou Y., Ding X., Wei H. (2022). Reproductive immune microenvironment. J. Reprod. Immunol..

[93] Chazara O., Xiong S., Moffett A. (2011). Maternal KIR and fetal HLA-C: A fine balance. J Leukoc. Biol..

[94] Choudhury R.H., Dunk C.E., Lye S.J., Harris L.K., Aplin J.D., Jones R.L. (2018). Decidual leucocytes infiltrating human spiral arterioles are rich source of matrix metalloproteinases and degrade extracellular matrix in vitro and in situ. Am. J. Reprod. Immunol..

[95] Nagamatsu T., Schust D.J. (2010). The immunomodulatory roles of macrophages at the maternal-fetal interface. Reprod. Sci..

[96] Soeters P.B., Grimble R.F. (2012). The conditional role of inflammation in pregnancy and cancer. Clin. Nutr..

[97] Firestein G.S., McInnes I.B. (2017). Immunopathogenesis of Rheumatoid Arthritis. Immunity.

[98] Wu Z.M., Yang H., Li M., Yeh C.C., Schatz F., Lockwood C.J., Di W., Huang S.J. (2012). Pro-inflammatory cytokine-stimulated first trimester decidual cells enhance macrophage-induced apoptosis of extravillous trophoblasts. Placenta.

[99] Alijotas-Reig J., Llurba E., Gris J.M. (2014). Potentiating maternal immune tolerance in pregnancy: A new challenging role for regulatory T cells. Placenta.

[100] Vento-Tormo R., Efremova M., Botting R., Turco M., Vento-Tormo M., Meyer K., Park J., Stephenson E., Polański K., Goncalves A. (2018). Single-cell reconstruction of the early maternal-fetal interface in humans. Nature.

[101] Liu S., Diao L., Huang C., Li Y., Zeng Y., Kwak-Kim J. (2017). The role of decidual immune cells on human pregnancy. J. Reprod. Immunol..

[102] Co E., Gormley M., Kapidzic M., Rosen D., Scott M., Stolp H., McMaster M., Lanier L., Bárcena A., Fisher S. (2013). Maternal decidual macrophages inhibit NK cell killing of invasive cytotrophoblasts during human pregnancy. Biol. Reprod..

[103] Smith S., Dunk C., Aplin J., Harris L., Jones R. (2009). Evidence for immune cell involvement in decidual spiral arteriole remodeling in early human pregnancy. Am. J. Pathol..

[104] Gomez-Lopez N., StLouis D., Lehr M., Sanchez-Rodriguez E., Arenas-Hernandez M. (2014). Immune cells in term and preterm labor. Cell. Mol. Immunol..

[105] Nancy P., Erlebacher A. (2014). T cell behavior at the maternal-fetal interface. Int. J. Dev. Biol..

[106] Wang W., Sung N., Gilman-Sachs A., Kwak-Kim J. (2020). T Helper (Th) Cell Profiles in Pregnancy and Recurrent Pregnancy Losses: Th1/Th2/Th9/Th17/Th22/Tfh Cells. Front. Immunol..

[107] Yang F., Zheng Q., Jin L. (2019). Dynamic Function and Composition Changes of Immune Cells During Normal and Pathological Pregnancy at the Maternal-Fetal Interface. Front. Immunol..

[108] Haseeb A., Haqqi T. (2013). Immunopathogenesis of osteoarthritis. Clin. Immunol..

[109] Asadi-Samani M., Bagheri N., Rafieian-Kopaei M., Shirzad H. (2017). Inhibition of Th1 and Th17 Cells by Medicinal Plants and Their Derivatives: A Systematic Review. Phytother. Res..

[110] Udalova I., Mantovani A., Feldmann M. (2016). Macrophage heterogeneity in the context of rheumatoid arthritis. Nat. Rev. Rheumatol..

[111] Zhu M., Yuan K., Lu Q., Zhu Q., Zhang S., Li X., Zhao L., Wang H., Luo G., Wang T. (2019). Emodin ameliorates rheumatoid arthritis by promoting neutrophil apoptosis and inhibiting neutrophil extracellular trap formation. Mol. Immunol..

[112] Li H., Hou Y., Zhang S., Zhou Y., Wang D., Tao S., Ni F. (2019). CD49a regulates the function of human decidual natural killer cells. Am. J. Reprod. Immunol..

[113] Wang W., Hao C., Lin Y., Yin G., Bao S., Qiu L., Lin Q. (2010). Increased prevalence of T helper 17 (Th17) cells in peripheral blood and decidua in unexplained recurrent spontaneous abortion patients. J. Reprod. Immunol..

[114] Zhao Q., Li Q., Fu Y., Ren C., Jiang A., Meng Y. (2022). Decidual macrophages in recurrent spontaneous abortion. Front. Immunol..

